# Decoupling of Bypass Efficiency and Mutagenicity of the 2-Acetylaminofluorene C8-Guanine Adduct by DNA Sequence Context

**DOI:** 10.3390/toxics14070620

**Published:** 2026-07-16

**Authors:** Yi-Tzai Chen, Rui Qi, Jian Ma, Ang Cai, Bongsup P. Cho, Deyu Li

**Affiliations:** Department of Biomedical and Pharmaceutical Sciences, College of Pharmacy, University of Rhode Island, Kingston, RI 02881, USA

**Keywords:** DNA damage, bulky adduct, conformational change, DNA replication, mutagenesis

## Abstract

DNA sequence context plays a critical role in modulating the mutational effects of DNA damage. Here, we investigated how base identity influences the replication bypass and mutagenicity of a site-specific dG-AAF (2-acetylaminofluorene) bulky adduct in the well-defined AG*N and TG*N sequence contexts. By selecting adenine and thymine as 5′-flanking bases and systematically varying the 3′-base, we established a controlled system to examine sequence-dependent lesion replication. We found that the 5′-flanking base strongly affects the bypass profile, with AG*N sequences exhibiting uniformly low bypass (≤9.7%) and TG*N sequences showing markedly elevated bypass (23.6–50.4%) with strong sequence dependence. These differences may arise from the structure of the dG-AAF, whose conformation heterogeneities are sensitive to the flanking sequence context. In contrast, mutagenicity remains consistently low across all sequences examined, with a low frequency of point mutations and no detectable frameshift events. These results reveal a clear decoupling between lesion bypass efficiency and replication fidelity, where sequence context strongly controls lesion tolerance but has limited impact on mutagenicity. In total, our findings demonstrate that DNA sequence affects lesion processing, providing insights into how local sequence context shapes genome stability and mutational processes.

## 1. Introduction

Genome damage leads to diverse biological outcomes and contributes to the development of disease, particularly cancer [1,2,3,4]. The sequence context surrounding a DNA lesion plays a critical role in determining its biological consequences [5,6,7,8]. It is well established that local sequence can influence the structural and conformational properties of bulky DNA lesions [9,10,11,12,13], which in turn affect how they are recognized and processed by cellular machinery. Variations in neighboring bases can alter base stacking, flexibility, and local geometry, thereby modulating the lesion’s conformational heterogeneity and, in turn, its accessibility to replication and repair enzymes [14]. As a result, the same chemical lesion can exhibit markedly different levels of replication bypass efficiency and mutagenicity depending on its sequence environment [15,16,17]. These observations underscore that the DNA sequence is not merely a passive background but an active player shaping the structural and functional consequences of DNA damage. Such sequence effects may also contribute to the formation of mutation hotspots and the emergence of characteristic mutational signatures observed in cancer genomes.

**Figure 1:**
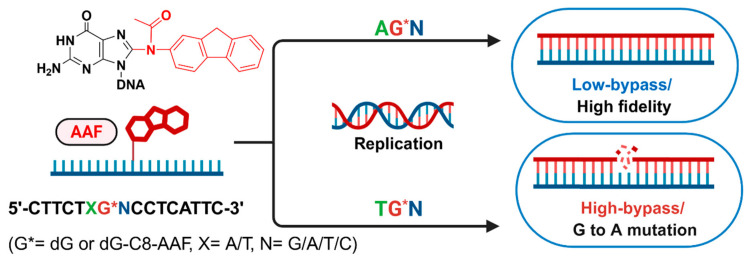
Chemical structure of C8 dG-AAF adduct and its biological effects in the AG*N and TG*N sequences (G* = dG-AAF).

Bulky DNA adducts formed from arylamine and nitroarene metabolites have long been studied for their roles in chemical carcinogenesis [18,19]. 2-Nitrofluorene is designated as a Group 2B carcinogen by the IARC [20]. Upon metabolic activation, 2-nitrofluorene is converted into a highly reactive nitrenium ion that covalently modifies DNA, predominantly forming C8-guanine adducts such as dG-AAF (*N*-(deoxyguanosin-8-yl)-2-acetylaminofluorene) (Figure 1) and the deacetylated dG-AF (*N*-(deoxyguanosin-8-yl)-2-aminofluorene) lesions [21,22,23,24,25]. dG-AAF and dG-AF are associated with an increased risk of bladder and liver cancers [18,19]. These bulky adducts are known to adopt sequence-dependent conformational ensembles, which influence their interactions with replication and repair machinery [14,26]. Functionally, dG-AF is largely non-mutagenic, whereas dG-AAF strongly impedes DNA polymerases and can induce both point mutations and frameshift events [24,25,27,28]. As a result, the dG-AAF lesion provides a powerful system for probing how structurally complex DNA damage is processed and how such processing contributes to mutagenesis and carcinogenesis.

In our previous studies, we systematically examined the replication bypass and mutagenicity of bulky arylamine adducts, focusing on the effects of cytosine methylation (m5C) on both bulky dG-AAF and dG-AF in the CG*N and m5CG*N sequence contexts [11,12]. The studies revealed that dG-AAF generally exhibits low bypass efficiency (<10%) but is highly sensitive to local sequence features. Notably, cytosine methylation introduced a striking context-dependent effect: m5C decreases bypass in 3′-pyrimidine contexts but increases it in 3′-purine contexts, compared to non-methylated cytosine sequences. In addition, dG-AAF was found to be frameshift-prone, with significant −G deletion mutations arising in specific sequence environments, highlighting a tight coupling between sequence context and mutagenic consequence. These findings established that even within a narrowly defined sequence context, local DNA sequence can strongly modulate both lesion tolerance and mutagenesis for bulky adducts.

While these epigenetics-related studies highlight the significant impact of m5C on lesion replication, an important question remains: what are the sequence effects in non-epigenetic sequences? To address this, we turned to sequence contexts defined solely by natural bases, focusing on AG*N and TG*N sequence contexts to examine the effects of A and T at the 5′ flanking position. In this study, we site-specifically modified eight 16-mer oligonucleotide sequences (d [5′-CTTCTXG*NCCTCATTC-3′], where X is either A or T, G* is either dG (control) or dG-AAF, and the 3′-flanking base (N) is A, T, C, or G). We then systematically conducted lesion bypass (Competitive Replication of Adduct Bypass, CRAB) and mutagenicity (Restriction Endonuclease And Post-labeling, REAP) experiments in a site-specific manner [29,30,31].

We found that DNA sequence context exerts strong control over lesion bypass, with the identity of the 5′-flanking base shifting the replication between low- and high-bypass groups (AG*N vs. TG*N). Within the TG*N group, the 3′-base further modulates the extent of bypass, revealing a hierarchical dependence on sequence. In contrast, mutagenicity remains consistently low across all contexts, indicating a clear distinction between replication efficiency (bypass) and fidelity (mutagenicity). These results show that local DNA sequences control lesion tolerance, even without relying on epigenetic changes. The systematic approach reveals the mechanism behind how sequence context shapes DNA damage processing, which likely influences where mutations occur across the genome.

## 2. Experimental Procedures

Lesion-containing oligonucleotides and vectors were prepared as previously described [11,29]. Eight 16mer oligonucleotides (5′-CTTCTXG*NCCTCATTC-3′, X = A/T, N = A/G/C/T) carrying a dG-AAF adduct were synthesized. The reactive N-acetoxy-N-(trifluoroacetyl)-2-acetylaminofluorene was prepared from 2-nitrofluorene via biomimetic activation and coupled to each oligonucleotide in sodium citrate buffer (pH 6.0, 37 °C, 24 h) [32]. After HPLC purification (>98% purity), the adducted strands were verified by LC-ESI-TOF-MS, and lesion location was confirmed by enzymatic digestion followed by MALDI-TOF/MS (Figures S1–S16 and Tables S1 and S2) [11,12].

Lesion-containing 16mers, unmodified 16mer controls, and 19mer competitor strands were phosphorylated, annealed with scaffolds and ligated into 58/61mer strands, which were inserted into the M13mp7(L2) ssDNA vector and verified by PCR (Figures S17–S21 and Table S3) [11].

**Figure 2:**
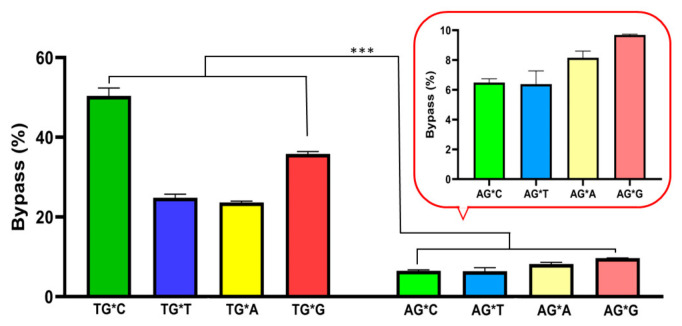
Bypass efficiencies of C8-dG-AAF under the TG*N (left) and AG*N (right) sequence contexts. Bypass efficiencies were determined using the CRAB assay in AlkB-negative *E. coli* cells. Data represent the mean (*n* = 3) ± standard deviation. Analyses of statistical significance were conducted by comparing the bypass efficiency of a certain sequence to the corresponding sequence in the other sequence group (such as TG*C vs. AG*C). All TG*N sequence contexts exhibit significantly higher bypass efficiencies than the corresponding AG*N sequence contexts (***, *p* < 0.001). The red inset shows an expanded view of the AGN series to facilitate comparison of the low-bypass sequence contexts.

**Figure 3:**
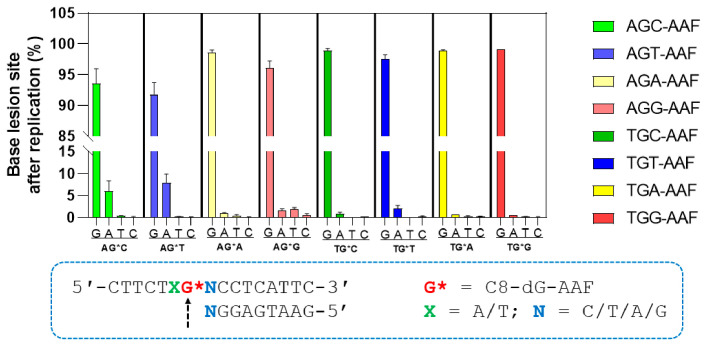
Mutagenic outcomes of G* (C8-dG-AAF) adduct in the HK82 *E. coli* cell (AlkB negative). The diagram (dotted line) shows the template containing G* and primer sequence, highlighting the incorporation point during replication. Mutation frequencies represent nucleotide incorporation opposite the dG-AAF lesion during replication. Data indicate no mutation (G) and point mutations (G to C/T/A). Data were generated from experiments in triplicate (the mean (*n* = 3) ± standard deviation). Different colors in the figure represent distinct sequence contexts and mutation groups.

Replication bypass (CRAB) and mutagenicity (REAP) assays were performed as previously described [11,29]. For replication bypass (CRAB) and mutagenicity (REAP) assays, lesion-bearing ssDNA was mixed with a lesion-free competitor genome (50:1 ratio) and electroporated into *Escherichia coli (E. coli*) HK82 (AlkB−) cells [30]. After phage amplification, DNA was isolated by QIAprep M13 kit (QIAGEN, Hilden, Germany), PCR-amplified, and double-digested with XhoI/SphI (New England Biolabs, Ipswich, MA, USA). And the resulting 20(CT)/28(AG)-mer (lesion) and 23-mer (competitor) fragments were analyzed by HPLC-ESI-TOF-MS in negative ion mode using a C18 column and an HFIP/methanol gradient. The competitor served as an internal reference for 100% bypass and zero induced mutation. Bypass efficiency was calculated as the lesion/competitor fragment intensity ratio normalized to the unmodified control (100%) (Figure 2, Table S4). Mutation frequencies were determined from the signal of each mutated sequence divided by total signals (Figure 3, Table S5) [11,12]. Data represent means ± SD (*n* = 3).

Statistical analyses of bypass efficiency and mutagenicity were performed as follows. The analyses were conducted by comparing the bypass efficiency or mutation of a certain sequence to either in a context group (either TG*N or AG*N group) or to the corresponding sequence in the other sequence group (such as TG*C vs. AG*C). The analyses were performed using IBM SPSS Statistics (Version 16.0) Differences in bypass efficiency among sequence contexts were assessed using Welch’s one-way ANOVA, followed by Games–Howell post hoc multiple-comparison tests. All tests were two-sided, and *p* values < 0.05 were considered statistically significant. ns: *p* > 0.05; * *p* < 0.05, ** *p* < 0.01, *** *p* < 0.001.

## 3. Results and Discussion

We measured both replication bypass efficiency and mutagenicity of the bulky dG-AAF using a site-specific approach, introducing a single dG-AAF adduct at a defined position: XG*N (X is either A or T, G* is either dG (control) or dG-AAF, and N is A, T, C, or G. Figure 1). The bypass assay quantified the ability of the polymerase to traverse the lesion, whereas the mutagenicity assay assessed replication fidelity. These complementary measurements distinguished whether sequence context primarily affects bypass efficiency, mutagenicity, or both, providing insight into how local DNA sequence controls the biological outcomes of DNA damage.

Selection of a single-stranded M13 vector and an AlkB-negative *E. coli* cell. To investigate the replication bypass efficiency and mutagenicity of the dG-AAF adduct, we used the CRAB and REAP assays with a single-stranded (ss) M13 vector [29]. This system minimizes interference from nucleotide excision repair (NER), since dG-AAF is repaired in double-stranded DNA. Using ss-DNA allows the cellular responses to show the intrinsic characteristics of the dG-AAF adduct without complications from NER. In this study, we used 1,*N*^6^-ethenoadenine (εA) as a control for the bypass and mutagenicity measurements. To minimize potential repair of εA by AlkB, we used an AlkB-negative *E. coli* strain (HK82) [31].

### 3.1. Bypass of dG-AAF in the AG*N Sequences

In the AG*N sequence series, lesion bypass exhibited only a modest dependence on the identity of the 3′ flanking base. Bypass efficiencies clustered within a relatively narrow range, from ~6.4% to ~9.7% (Figure 2). Specifically, AG*C (6.5%) and AG*T (6.4%) showed nearly identical, lower bypass levels; whereas AG*A (8.2%) and AG*G (9.7%) exhibited slightly higher bypass efficiencies. This yields a sequence-dependent bypass efficiency of AG*G > AG*A > AG*C ≈ AG*T, with moderate variations.

### 3.2. Bypass of dG-AAF in the TG*N Sequences

In the TG*N sequence series, lesion bypass exhibited a pronounced dependence on the identity of the 3′ flanking base. Bypass efficiencies varied widely, from ~23.6% to ~50.4%. Among the four sequences, TG*C showed the highest bypass (~50.4%), followed by TG*G (~35.8%); whereas TG*T (~24.8%) and TG*A (~23.6%) displayed substantially lower and nearly identical bypass levels. These data establish a sequence-dependent order of TG*C > TG*G > TG*T ≈ TG*A. Notably, the ′ efficiency in the TG*N context. These results demonstrate that, when the lesion is preceded by 5′ T, the 3′ flanking nucleotide becomes a greater determining factor of bypass efficiency than A.

### 3.3. Comparison of Bypass Between AG*N and TG*N Sequences

Overall, the AG*N and TG*N series exhibit strikingly different bypass behaviors. The AG*N sequences show low and relatively uniform bypass efficiencies (average ~7.7%), with modest variation across the four 3′-flanking bases. In contrast, the TG*N sequences display substantially higher bypass (average ~33.7%, ~4.4-fold greater than AG*N) with a stronger dependence on 3′ base identity. These results show that the 5′-flanking base strongly modulates the extent of dG-AAF lesion bypass.

### 3.4. Comparison of Bypass Between AG*N/TG*N to CG*N/m5CG*N

The bypass patterns of dG-AAG observed in the AG*N and TG*N series differ substantially from those previously reported for the CG*N/m5CG*N sequence contexts. In the CG*N/m5CG*N study [12], bypass efficiencies were uniformly low (<10%) and exhibited a clear sequence-dependent inversion upon cytosine methylation, with m5C decreasing bypass in C^#^GC and C^#^GT but increasing bypass in C^#^GA and C^#^GG (C^#^ = either C or m5C). A similar low-bypass group is observed in the AG*N series, where bypass remains uniformly low (maximum ~9.7%) and varies modestly across the four 3′ flanking bases. In contrast, the TG*N series shows a dramatically elevated bypass rate, ranging from 23.6% to 50.4%, representing a substantial increase over both the AG*N and CG*N/m5CG*N contexts. These results suggest that the 5′ flanking base plays an important role in modulating both the magnitude and sequence dependence of dG-AAF bypass.

One possible explanation for the elevated bypass observed in the TG*N series is that thymine creates a less sterically crowded environment than adenine adjacent to the bulky dG-AAF lesion. However, previous studies [9,32] have shown that dG-AAF adopts sequence-dependent conformational ensembles that are highly sensitive to neighboring bases. Therefore, the observed differences likely reflect a combination of local steric effects and sequence-dependent conformational changes in the lesion-containing DNA.

We performed statistical analyses to evaluate differences in bypass efficiency among the various sequence contexts. The TG*N series exhibited significantly higher bypass efficiencies than the AG*N series across all sequence contexts (*p* < 0.001; Figure 2 and Table S6). Within the TG*N series, TG*C displayed the highest bypass efficiency and differed significantly from the other three sequence contexts, whereas TG*G exhibited a significantly higher bypass than TG*A and TG*T (Table S6). No significant difference was observed between TG*A and TG*T. In the AG*N series, AG*G showed significantly higher bypass than AG*C (*p* < 0.01), while no significant differences were detected among AG*A, AG*C, and AG*T. Detailed statistical comparisons are provided in Table S6.

### 3.5. Mutagenicity of dG-AAF in AG*N and TG*N Sequences

The mutagenicity of dG-AAF in the AG*N and TG*N sequence contexts was relatively low and exhibited limited overall sequence dependence (Figure 3). Across all sequences, correct G incorporation predominated, ranging from 91.8% (AG*T) to 99.1% (TG*G), indicating largely error-free replication. Consistent with this, most point mutations occurred at very low frequencies, with G→C mutations ≤0.5% and G→T mutations ≤1.9% across all sequence contexts. The G→A mutations displayed a sequence-dependent bias in the AG*N series, being elevated in 3′ pyrimidine contexts (6.0% for AG*C and 7.9% for AG*T) but remaining low in 3′ purine contexts (0.9% for AG*A and 1.6% for AG*G). Importantly, no −G deletion mutations were detected in any AG*N or TG*N sequence, contrary to our previous observations of the -G deletion in the CG*N/m5CG*N sequences [12]. Overall, these results indicate that, despite substantial sequence-dependent differences in bypass efficiency, mutagenicity remains low. However, specific substitution patterns, such as G→A, retain a measurable sequence dependence modulated by local base identity.

We also performed statistical analyses of the total mutation frequencies, defined as the sum of G→A, G→T, and G→C mutations, across all sequence contexts. In contrast to the bypass results, no significant differences in total mutation frequency were observed between the AG*N and TG*N series (Table S7). Similarly, no statistically significant differences were detected among individual sequence contexts within either the AG*N or TG*N groups (all *p* > 0.05; Table S7). These results indicate that, despite substantial sequence-dependent differences in bypass efficiency, the overall mutagenicity of the dG-AAF lesion remains similar across the sequence contexts examined.

### 3.6. Comparison of Mutagenicity of dG-AAF in AG*N/TG*N Versus CG*N/m5CG*N Sequence Contexts

In contrast to our previous findings in the CG*N/m5CG*N sequence contexts [12], where dG-AAF induced substantial sequence-dependent mutagenesis, the AG*N and TG*N series exhibited markedly different behavior. In the CG*N/m5CG*N study, frameshift mutations (-G deletion) were a major mutational outcome, particularly in the CG*A, CG*G, m5CG*A, and m5CG*G sequence contexts. However, these −G deletion mutations were not observed uniformly across all sequence contexts, as little or no frameshift mutagenesis was detected in CG*C, CG*T, m5CG*C, or m5CG*T. In the present study, no −G deletion mutations were detected in any AG*N or TG*N sequence, and overall mutagenicity remained low across all contexts. While minor sequence-dependent variations in point mutations were observed, most notably a modest increase in G→A substitutions in AG*C and AG*T, these effects were limited in magnitude and did not approach the mutational impact observed in the CG*A, CG*G, m5CG*A, and m5CG*G contexts, where −G deletion frequencies reached as high as 45.9%. Together, these findings indicate that frameshift mutagenesis is highly sequence-specific rather than an intrinsic property of the dG-AAF lesion itself. The molecular basis for this selectivity remains unclear but may involve sequence-dependent conformational states that differentially favor slipped mutagenic intermediates during replication [32].

### 3.7. Overall Comparison of dG-AAF in AG*N and TG*N Sequence Contexts

The AG*N and TG*N sequence series exhibit both similarities and key differences in lesion processing. In both contexts, mutagenicity remains uniformly low, with correct G incorporation predominating (>90–99%), minimal point mutations (G→C ≤0.5%, G→T ≤1.9%), and no −G deletion observed, indicating largely error-free replication. In contrast, their bypass behaviors diverge substantially. The AG*N series displays a low-bypass group (maximum ~9.7%) with modest variation across the four 3′ bases, whereas the TG*N series shows markedly elevated bypass (23.6–50.4%) with strong sequence dependence, spanning more than a two-fold range. Thus, while both contexts support high-fidelity replication, they differ markedly in bypass efficiency. These findings reveal a clear decoupling between bypass and mutagenicity, in which sequence context strongly affects lesion-bypass efficiency but has minimal impact on replication fidelity in AG*N and TG*N sequences. Statistical analyses reinforced these observations. The TG*N series exhibited significantly higher bypass efficiencies than the AG*N series (Figure 2 and Table S6), whereas total mutation frequencies remained statistically indistinguishable between groups and among individual sequence contexts (Table S7). These findings provide quantitative evidence that sequence context exerts a much stronger influence on lesion bypass than on mutagenicity, supporting a functional separation between lesion tolerance and replication fidelity.

The substantial difference in bypass efficiency between the AGN and TGN sequence contexts suggests that local DNA sequence can strongly influence lesion tolerance during replication. Although both sequence groups exhibited similarly low mutagenicity, the TGN sequences were bypassed much more efficiently than the AGN sequences. Consequently, lesions in different sequence contexts may have markedly different effects on replication progression despite producing similar mutational outcomes. These findings indicate that mutation frequency alone may not fully capture the biological consequences of DNA damage and highlight the importance of considering both lesion bypass and mutagenicity when evaluating sequence-dependent lesion processing.

### 3.8. Limitations of the E. coli Model System

This study was conducted in an *E. coli* system using an M13 single-stranded vector to minimize the influence of DNA repair and to directly assess the intrinsic bypass and mutagenic properties of dG-AAF in defined sequence contexts. While this system provides a powerful and controlled platform for mechanistic analysis, it also has inherent limitations. Lesion processing in double-stranded vector and in mammalian cells is likely to be influenced by additional factors, including nucleotide excision repair, translesion synthesis DNA polymerases, chromatin organization, and cell-type-specific DNA damage responses. Consequently, the quantitative bypass efficiencies and mutagenic outcomes reported here may differ from those observed in more complex biological systems.

## 4. Conclusions

In this study, we systematically investigated how local DNA sequence context impacts the replication block and mutagenic property of a site-specific C8 dG-AAF lesion. The results reveal a clear functional separation between lesion bypass and mutagenesis. These findings demonstrate sequence-dependent lesion replication in which local DNA sequence (AG*N vs. TG*N) determines both the magnitude and sensitivity of lesion bypass. The identity of the 5′ flanking base defines the bypass group, shifting the system between low- and high-tolerance states, while the 3′ flanking base modulates variation within that group. Notably, sequence context strongly influences replication efficiency without substantially altering fidelity, demonstrating a clear functional separation between lesion tolerance and mutagenesis. Compared with our previous CG*N/m5CG*N study [12], where epigenetic modification drives context-dependent replication, the present work shows that changing the identity of the 5′ flanking base from A to T can strongly influence dG-AAF bypass efficiency. Notably, the TG*N series exhibits substantially higher bypass than the corresponding AG*N series. The dG-AAF results suggest that local DNA sequence inherently influences how bulky DNA lesions is processed during replication. These findings further support the idea that local DNA sequence can influence how lesions are tolerated and propagated during replication. While we did not directly probe lesion conformation in this study, our results are consistent with prior structural studies that have shown sequence-dependent conformational heterogeneity of the dG-AAF adducts [11,12,26].

Our recent work [11,12] establishes an integrated system in which local DNA sequence context affects the processing of bulky DNA lesions at multiple levels and demonstrates how epigenetic modification (m5C) modulates lesion bypass and mutagenicity in a sequence-dependent manner. In the present paper, we show that natural base identity alone can exert strong control as well, with the 5′ flanking base defining the global bypass regime and the 3′ base tuning local variation, while mutagenicity remains largely uncoupled. These findings provide a model system for understanding how sequence context shapes genome stability and mutation patterns.

This work opens several possibilities for future investigation. Extending these studies to human cells with diverse repair pathways will be important to evaluate the interplay between sequence effects and cellular repair/replication mechanisms. In addition, expanding the sequence beyond the 5′ and 3′ neighboring bases and lesion types may help define general rules governing sequence-dependent lesion processing across different DNA damage types. Finally, integrating these insights with genomic and cancer datasets could reveal how sequence-related lesion tolerance contributes to mutation hotspots and mutational signatures, linking molecular mechanisms to genome-wide patterns of mutagenesis.

## Data Availability

The original contributions presented in this study are included in the article/Supplementary Materials. Further inquiries can be directed to the corresponding authors.

## Supplementary Materials

The following supporting information can be downloaded at: https://www.mdpi.com/article/10.3390/toxics14070620/s1. Figure S1. ESI-TOF analysis of 16mer oligo containing AG*C (G* = dG-C8-AAF); Figure S2. ESI-TOF analysis of 16mer oligo containing TG*C (G* = dG-C8-AAF); Figure S3. ESI-TOF analysis of 16mer oligo containing AG*T (G* = dG-C8-AAF); Figure S4. ESI-TOF analysis of 16mer oligo containing TG*T (G* = dG-C8-AAF); Figure S5. ESI-TOF analysis of 16mer oligo containing AG*A (G* = dG-C8-AAF); Figure S6. ESI-TOF analysis of 16mer oligo containing TG*A (G* = dG-C8-AAF); Figure S7. ESI-TOF analysis of 16mer oligo containing AG*G (G* = dG-C8-AAF); Figure S8. ESI-TOF analysis of 16mer oligo containing TG*G (G* = dG-C8-AAF); Figure S9. MALDI-TOF mass spectra of 16mer oligo containing AG*C (G* = dG-C8-AAF); Figure S10. MALDI-TOF mass spectra of 16mer oligo containing TG*C (G* = dG-C8-AAF); Figure S11. MALDI-TOF mass spectra of 16mer oligo containing AG*T (G* = dG-C8-AAF); Figure S12. MALDI-TOF mass spectra of 16mer oligo containing TG*T (G* = dG-C8-AAF); Figure S13. MALDI-TOF mass spectra of 16mer oligo containing AG*A (G* = dG-C8-AAF); Figure S14. MALDI-TOF mass spectra of 16mer oligo containing TG*A (G* = dG-C8-AAF); Figure S15. MALDI-TOF mass spectra of 16mer oligo containing AG*G (G* = dG-C8-AAF); Figure S16. MALDI-TOF mass spectra of 16mer oligo containing TG*G (G* = dG-C8-AAF); Figure S17. Diagram of construction of 58mer lesion containing oligonucleotide using the AG*C sequence as an illustration (G* = dG-C8-AAF); Figure S18. Denaturing urea polyacrylamide gel of 58mer lesion containing oligonucleotide using the AG*C sequence as an illustration (G* = dG-C8-AAF); Figure S19. Diagram of PCR analysis for lesion containing M13 genome using the AG*C sequence as an illustration (G* = dG-C8-AAF); Figure S20. Diagram of 15% polyacrylamide gel of PCR products of lesion containing M13 genome using the AG*C sequence with dG-AAF as an illustration; Figure S21. Diagram of the REAP & CRAB procedures using AG*N as an illustration (G* = dG-C8-AAF); Table S1. Calculated and observed monoisotopic MW and m/z value of modified oligonucleotides. (G* = dG-C8-AAF); Table S2. Calculated and observed monoisotopic mass values and m/z values measured by MALDI-TOF; Table S3. List of oligonucleotide and primer sequences (5′ to 3′) used for the REAP and CRAB assays; Table S4. Results of bypass of dG-AAF in HK82 *E. coli* cell (AlkB-); Table S5. Results of mutagenicity of dG-AAF in HK82 *E. coli* cell (AlkB-); Table S6. Statistical analyses of bypass efficiencies; Table S7. Statistical analyses of total mutations.
