# Supplementary material for: Decoupling of Bypass Efficiency and Mutagenicity of the 2-Acetylaminofluorene C8-Guanine Adduct by DNA Sequence Context
Source: Toxics. 2026 Jul 16;14(7):620. doi: 10.3390/toxics14070620 (PMC13417602; doi:10.3390/toxics14070620)
Supplement: Supplementary file 1 [file toxics-14-00620-s001.zip › toxics-4356315-supplementary.pdf]

SUPPORTING INFORMATION

Decoupling of Bypass Efficiency and Mutagenicity of the 2-Acetylaminofluorene C8-Guanine Adduct by DNA Sequence Context

Yi-Tzai Chen, Rui Qi, Jian Ma, Ang Cai, Bongsup P. Cho*, and Deyu Li*

Department of Biomedical and Pharmaceutical Sciences, College of Pharmacy, University of Rhode Island, Kingston, RI, 02881, United States of America

* To whom correspondence should be addressed.

Tel: +1 (401) 874-9361 (DL); +1 (401) 874-5024 (BPC).

Email: deyuli@uri.edu (DL); bcho@uri.edu (BPC).

Table of Contents

Figure S1. ESI-TOF analysis of 16mer oligo containing AG*C (G*=dG-C8-AAF).

Figure S2. ESI-TOF analysis of 16mer oligo containing TG*C (G*=dG-C8-AAF).

Figure S3. ESI-TOF analysis of 16mer oligo containing AG*T (G*=dG-C8-AAF).

Figure S4. ESI-TOF analysis of 16mer oligo containing TG*T (G*=dG-C8-AAF).

Figure S5. ESI-TOF analysis of 16mer oligo containing AG*A (G*=dG-C8-AAF).

Figure S6. ESI-TOF analysis of 16mer oligo containing TG*A (G*=dG-C8-AAF).

Figure S7. ESI-TOF analysis of 16mer oligo containing AG*G (G*=dG-C8-AAF).

Figure S8. ESI-TOF analysis of 16mer oligo containing TG*G (G*=dG-C8-AAF).

Figure S9. MALDI-TOF mass spectra of 16mer oligo containing AG*C (G*=dG-C8-AAF).

Figure S10. MALDI-TOF mass spectra of 16mer oligo containing TG*C (G*=dG-C8-AAF).

Figure S11. MALDI-TOF mass spectra of 16mer oligo containing AG*T (G*=dG-C8-AAF).

Figure S12. MALDI-TOF mass spectra of 16mer oligo containing TG*T (G*=dG-C8-AAF).

Figure S13. MALDI-TOF mass spectra of 16mer oligo containing AG*A (G*=dG-C8-AAF).

Figure S14. MALDI-TOF mass spectra of 16mer oligo containing TG*A (G*=dG-C8-AAF).

Figure S15. MALDI-TOF mass spectra of 16mer oligo containing AG*G (G*=dG-C8-AAF).

Figure S16. MALDI-TOF mass spectra of 16mer oligo containing TG*G (G*=dG-C8-AAF).

Figure S17. Diagram of construction of 58mer lesion containing oligonucleotide using the AG*C sequence as an illustration (G*=dG-C8-AAF).

Figure S18. Denaturing urea polyacrylamide gel of 58mer lesion containing oligonucleotide using the AG*C sequence as an illustration (G*=dG-C8-AAF).

Figure S19. Diagram of PCR analysis for lesion containing M13 genome using the AG*C sequence as an illustration (G*=dG-C8-AAF).

Figure S20. Diagram of 15% polyacrylamide gel of PCR products of lesion containing M13 genome using the AG*C sequence with dG-AAF as an illustration.

Figure S21. Diagram of the REAP & CRAB procedures using AG*N as an illustration (G*=dG-C8-AAF).

Table S1. Calculated and observed monoisotopic MW and m/z value of modified oligonucleotides. (G*=dG-C8-AAF).

Table S2. Calculated and observed monoisotopic mass values and m/z values measured by MALDI-TOF.

Table S3. List of oligonucleotide and primer sequences (5′ to 3′) used for the REAP and CRAB assays.

Table S4. Results of bypass of dG-AAF in HK82 E. coli cell (AlkB-).

Table S5. Results of mutagenicity of dG-AAF in HK82 E. coli cell (AlkB-).

Table S6. Statistical analyses of bypass efficiencies.

Table S7. Statistical analyses of total mutations.

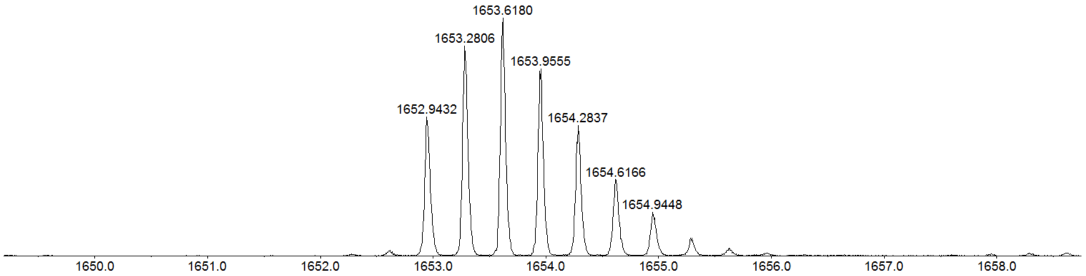

Figure S1. ESI-TOF analysis of 16mer oligo containing AG*C (G*=dG-C8-AAF). Data represent the −3 charge envelope.

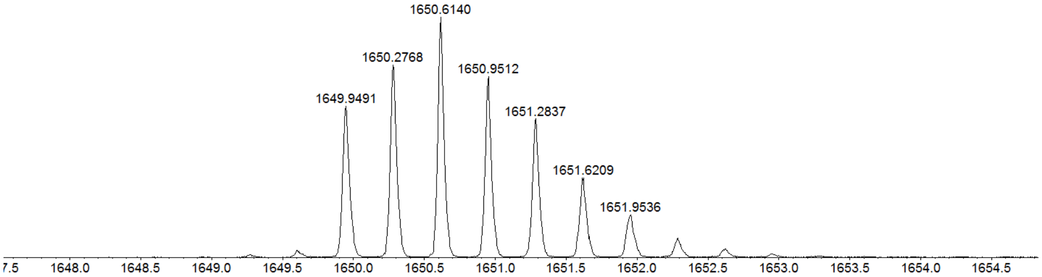

Figure S2. ESI-TOF analysis of 16mer oligo containing TG*C (G*=dG-C8-AAF). Data represent the −3 charge envelope.

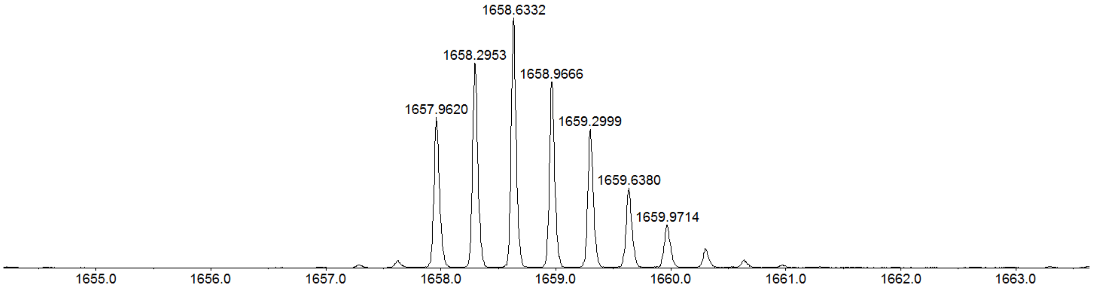

Figure S3. ESI-TOF analysis of 16mer oligo containing AG*T (G*=dG-C8-AAF). Data represent the −3 charge envelope.

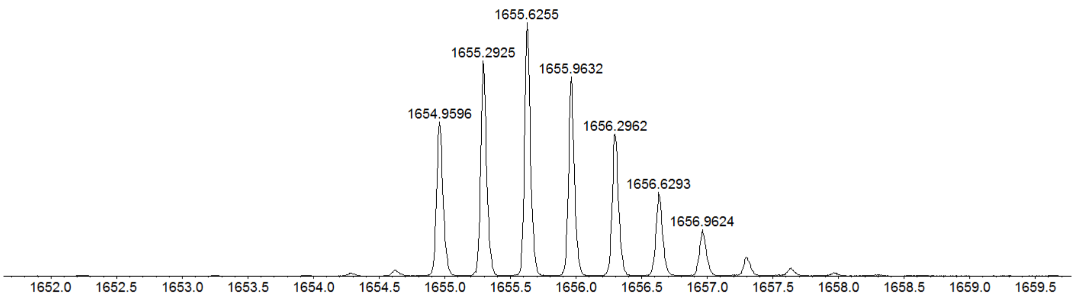

Figure S4. ESI-TOF analysis of 16mer oligo containing TG*T (G*=dG-C8-AAF). Data represent the −3 charge envelope.

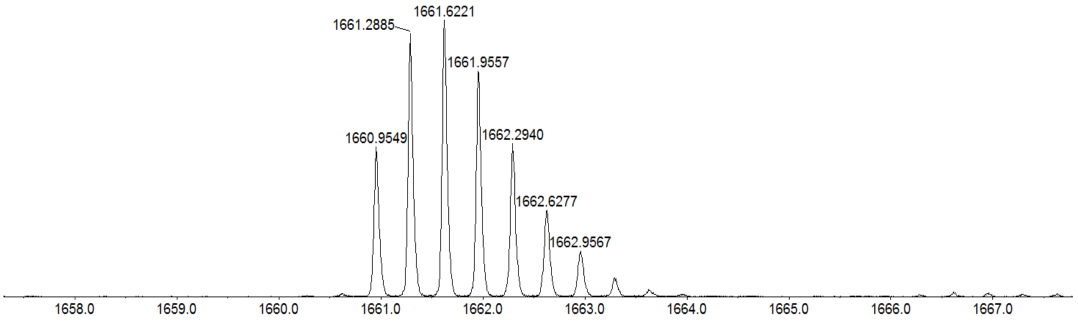

Figure S5. ESI-TOF analysis of 16mer oligo containing AG*A (G*=dG-C8-AAF). Data represent the −3 charge envelope.

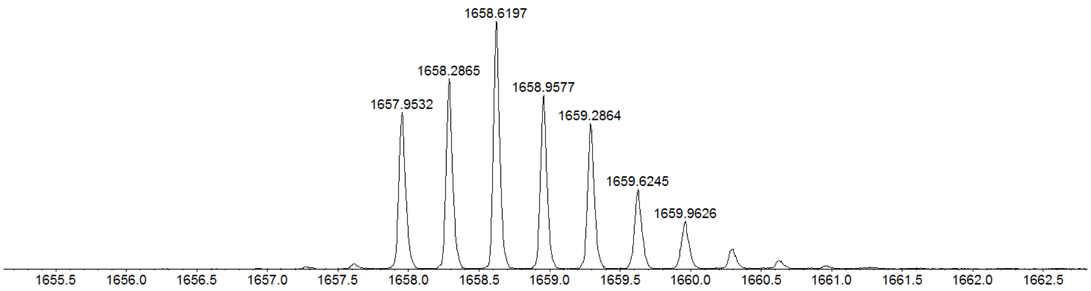

Figure S6. ESI-TOF analysis of 16mer oligo containing TG*A (G*=dG-C8-AAF). Data represent the −3 charge envelope.

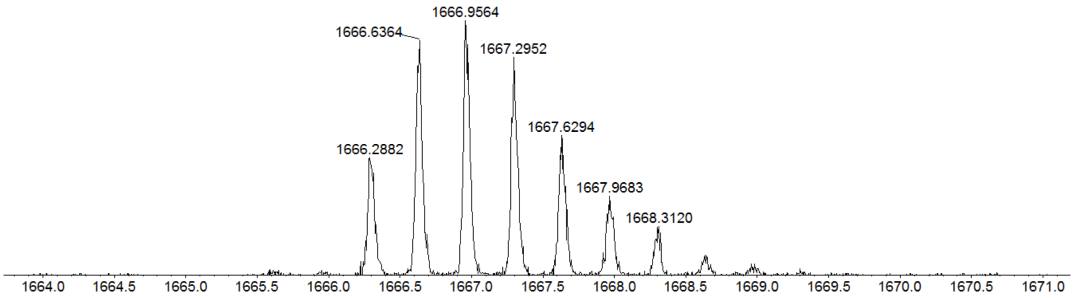

Figure S7. ESI-TOF analysis of 16mer oligo containing AG*G (G*=dG-C8-AAF). Data represent the −3 charge envelope.

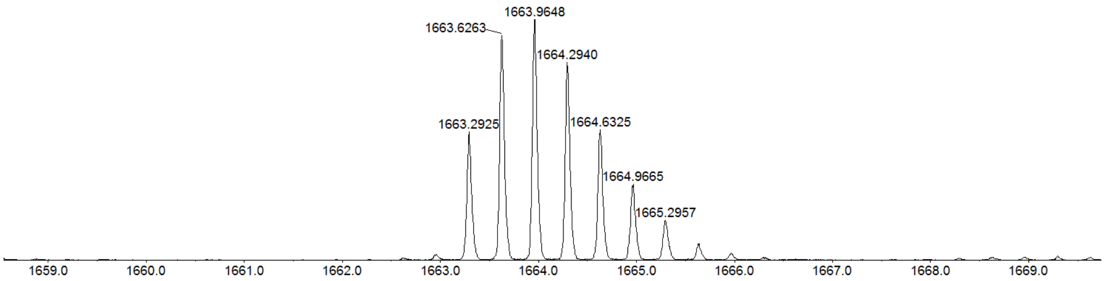

Figure S8. ESI-TOF analysis of 16mer oligo containing TG*G (G*=dG-C8-AAF). Data represent the −3 charge envelope.

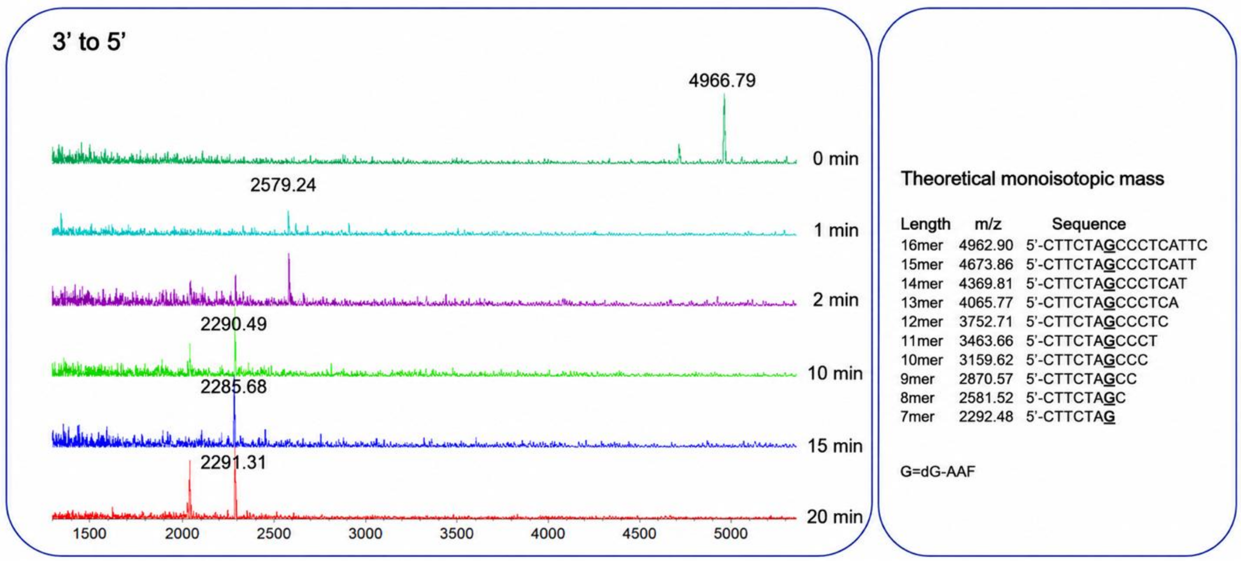

Figure S9. MALDI-TOF mass spectra of 16mer oligo containing AG*C (G*=dG-C8-AAF). 3′→5′ exonuclease digestions (SVP enzyme) in Reflectron mode are shown at various time intervals. Insets provide theoretical m/z of the corresponding fragments.

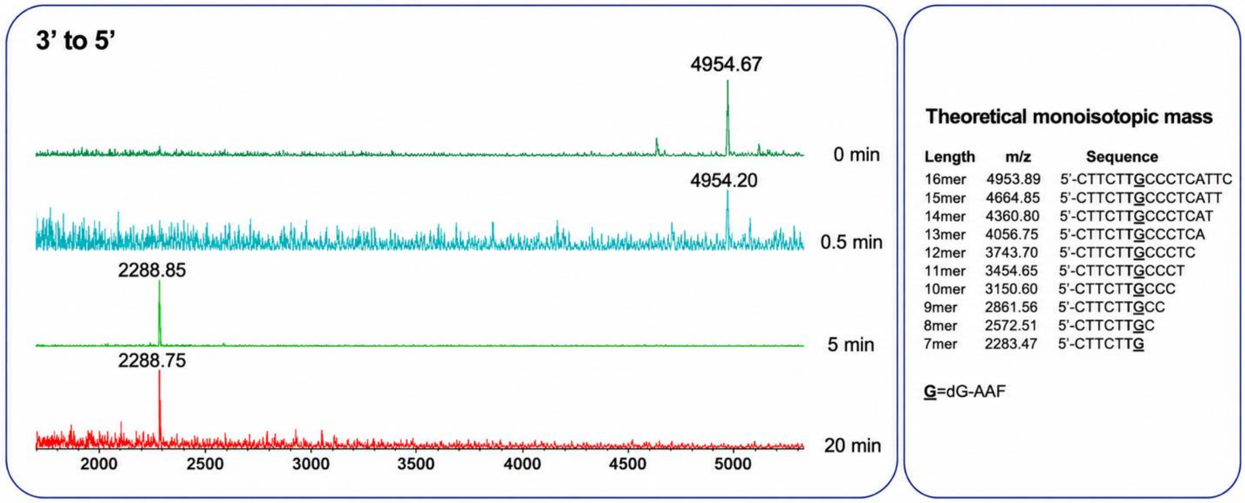

Figure S10. MALDI-TOF mass spectra of 16mer oligo containing TG*C (G*=dG-C8-AAF). 3′→5′ exonuclease digestions (SVP enzyme) in Reflectron mode are shown at various time intervals. Insets provide theoretical m/z of the corresponding fragments.

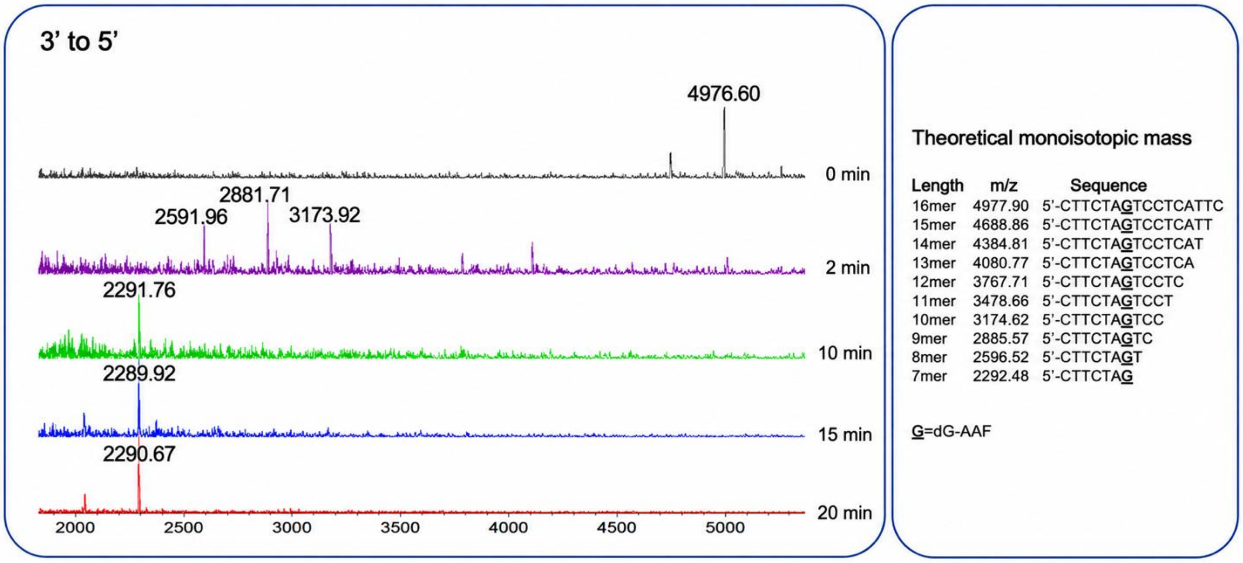

Figure S11. MALDI-TOF mass spectra of 16mer oligo containing AG*T (G*=dG-C8-AAF). 3′→5′ exonuclease digestions (SVP enzyme) in Reflectron mode are shown at various time intervals. Insets provide theoretical m/z of the corresponding fragments.

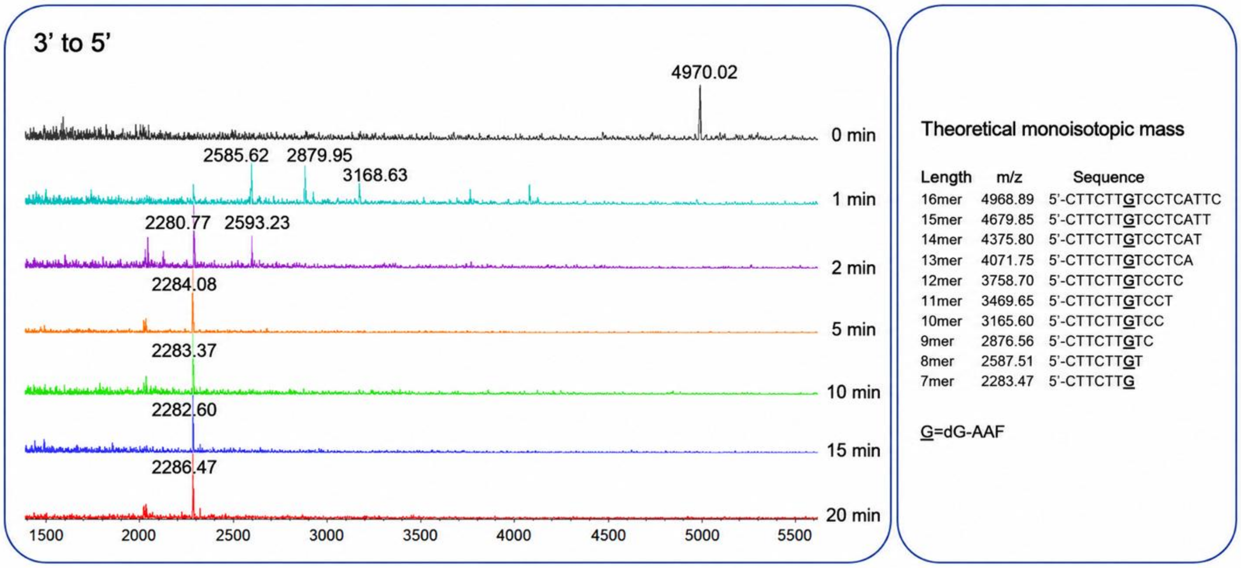

Figure S12. MALDI-TOF mass spectra of 16mer oligo containing TG*T (G*=dG-C8-AAF). 3′→5′ exonuclease digestions (SVP enzyme) in Reflectron mode are shown at various time intervals. Insets provide theoretical m/z of the corresponding fragments.

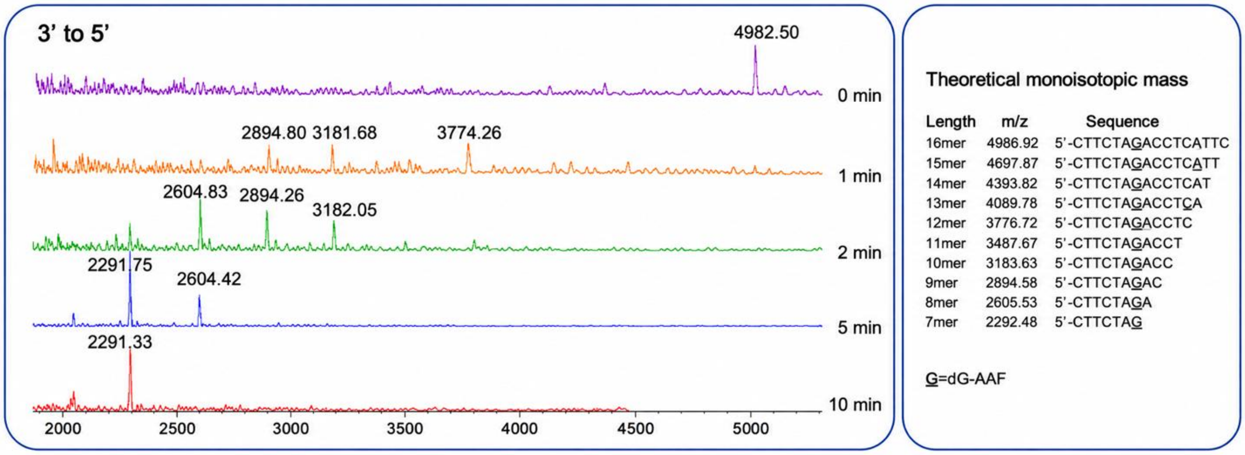

Figure S13. MALDI-TOF mass spectra of 16mer oligo containing AG*A (G*=dG-C8-AAF). 3′→5′ exonuclease digestions (SVP enzyme) in Reflectron mode are shown at various time intervals. Insets provide theoretical m/z of the corresponding fragments.

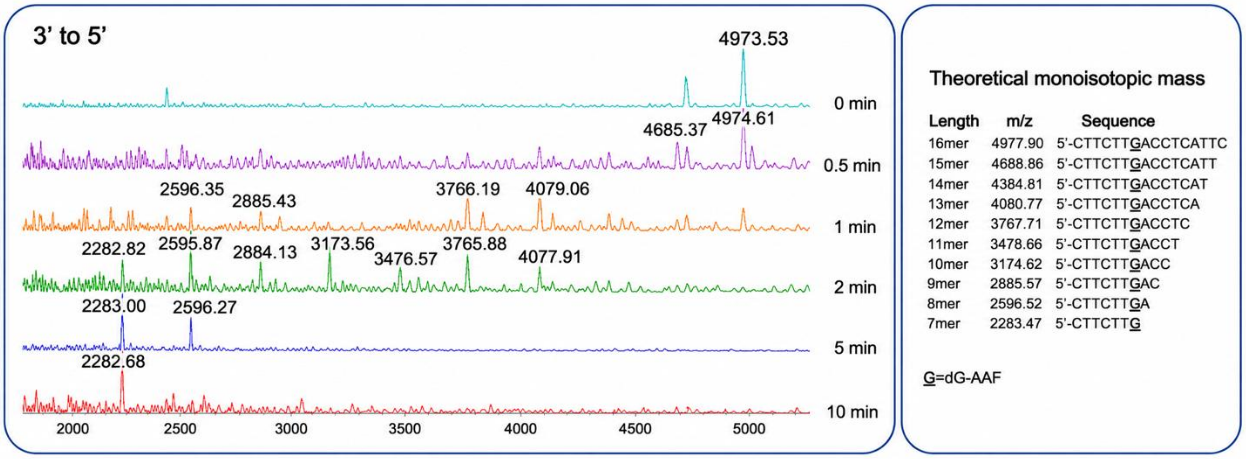

Figure S14. MALDI-TOF mass spectra of 16mer oligo containing TG*A (G*=dG-C8-AAF). 3′→5′ exonuclease digestions (SVP enzyme) in Reflectron mode are shown at various time intervals. Insets provide theoretical m/z of the corresponding fragments.

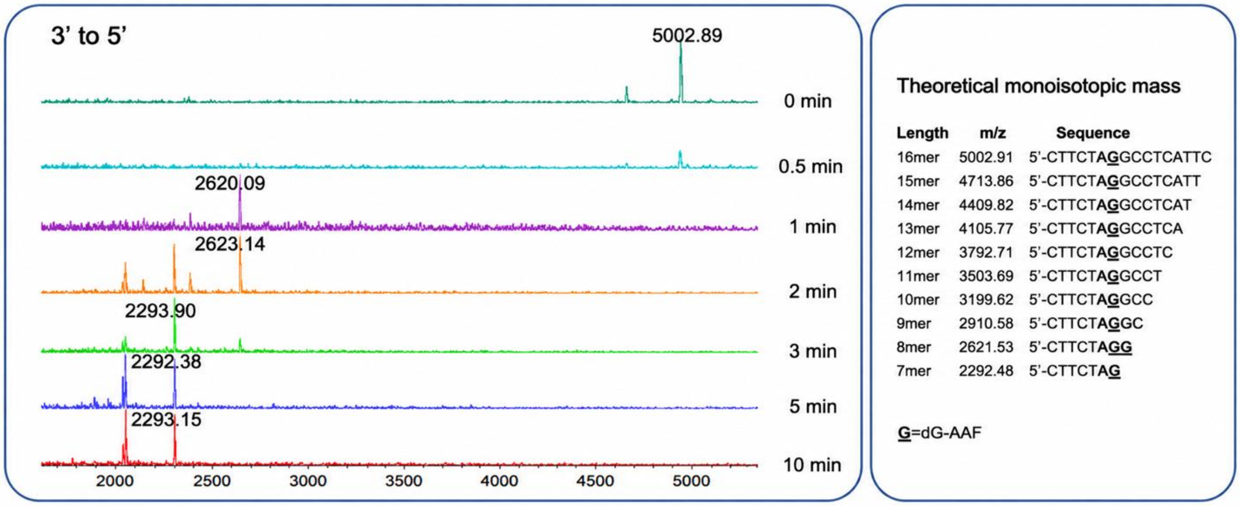

Figure S15. MALDI-TOF mass spectra of 16mer oligo containing AG*G (G*=dG-C8-AAF). 3′→5′ exonuclease digestions (SVP enzyme) in Reflectron mode are shown at various time intervals. Insets provide theoretical m/z of the corresponding fragments.

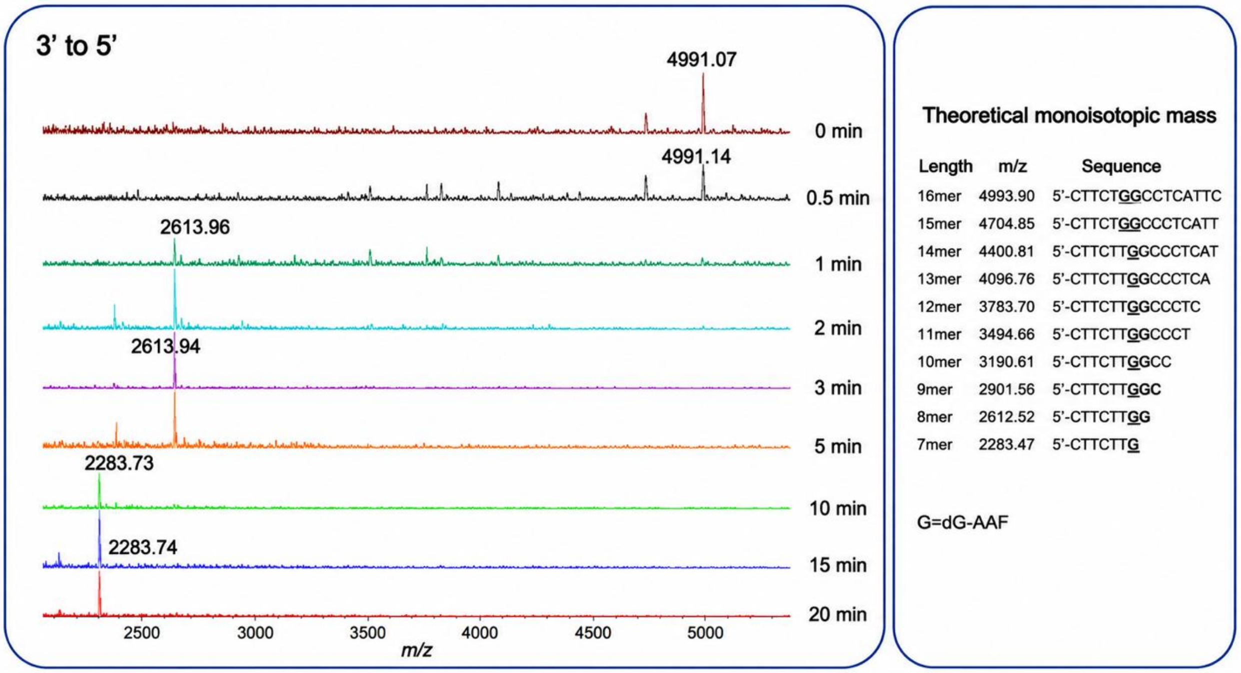

Figure S16. MALDI-TOF mass spectra of 16mer oligo containing TG*G (G*=dG-C8-AAF). 3′→5′ exonuclease digestions (SVP enzyme) in Reflectron mode are shown at various time intervals. Insets provide theoretical m/z of the corresponding fragments.

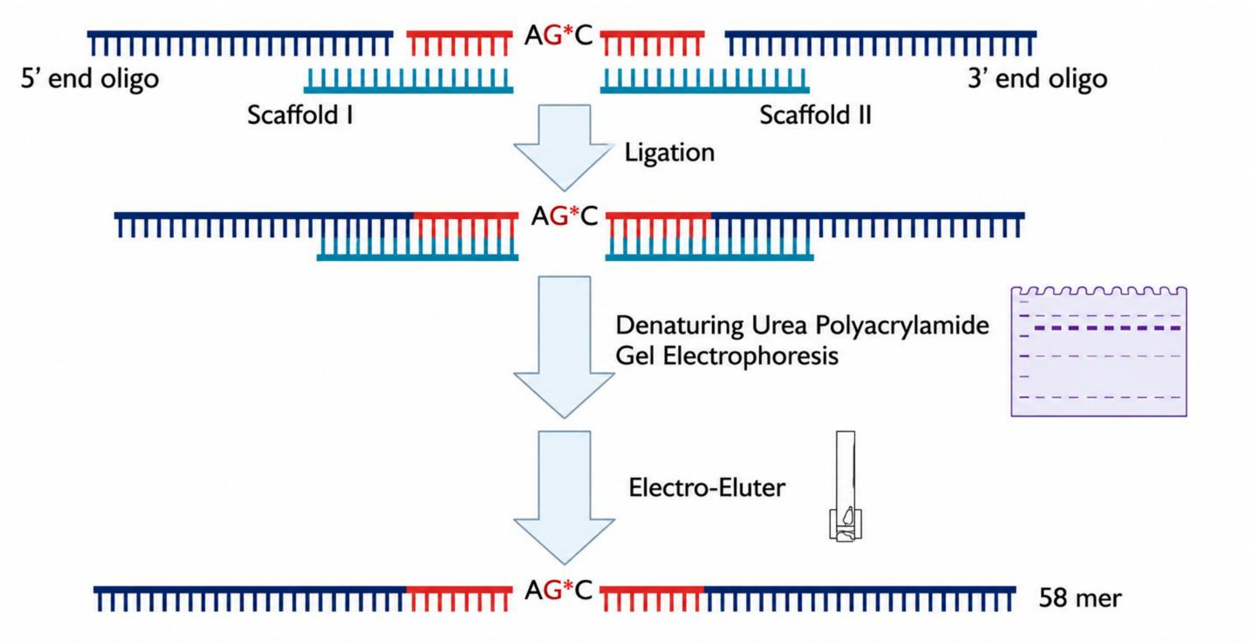

Figure S17. Diagram of the construction of 58mer lesion containing oligonucleotide using the AG*C sequence as an illustration (G*=dG-C8-AAF).

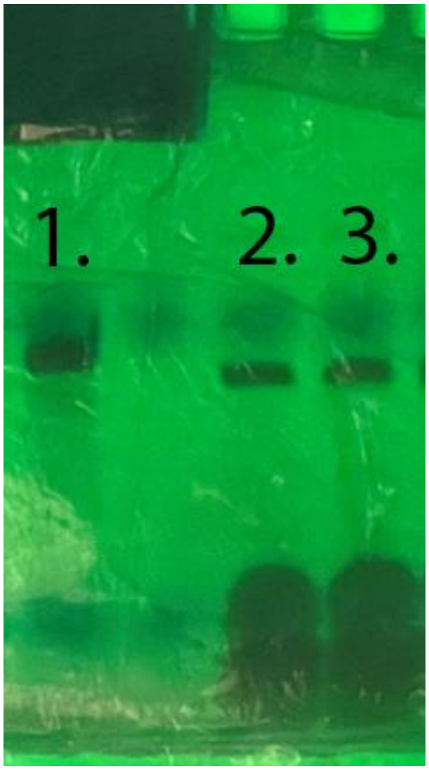

Figure S18. Denaturing urea polyacrylamide gel (PAGE) of the 58mer lesion containing oligonucleotide using the AG*C sequence as an illustration (G*=dG-C8-AAF). Lane 1, 58mer Control. Lane 2-3, 58mer AG*C ligation product.

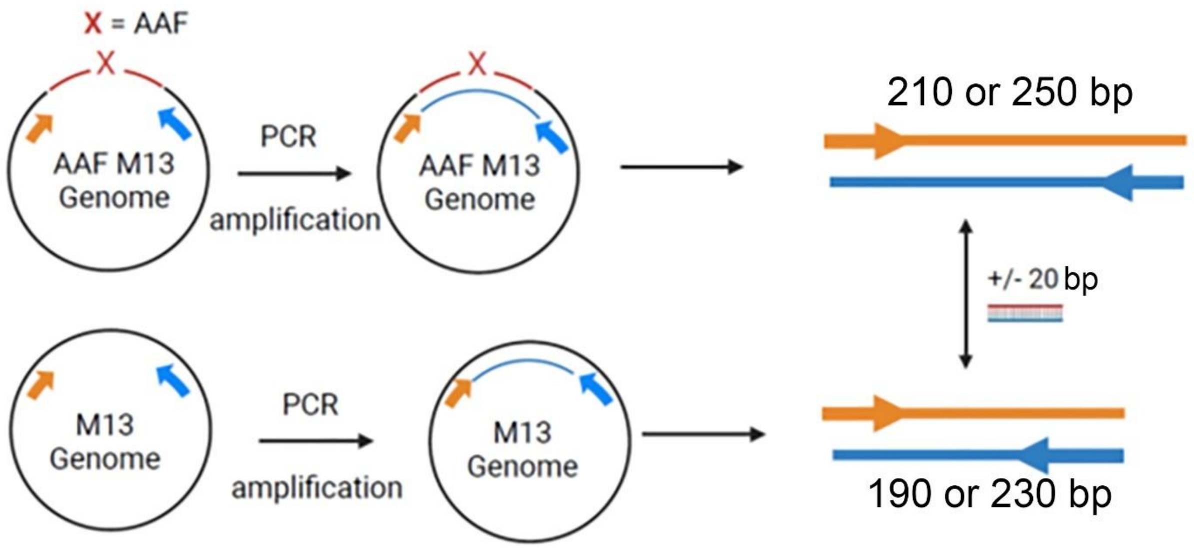

Figure S19. Diagram of PCR analysis for lesion containing M13 genome using the dG-AAF containing sequence as an illustration (X = dG-C8-AAF).

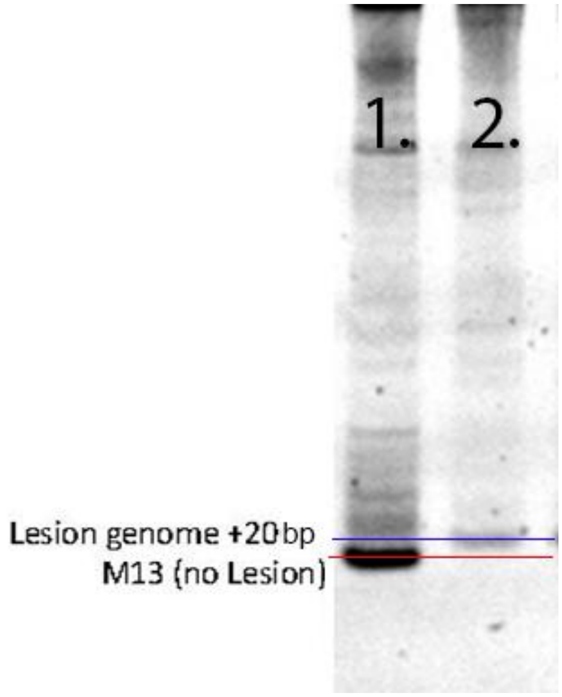

Figure S20. Diagram of 15% polyacrylamide gel of PCR products of lesion containing M13 genome using dG-AAF containing sequence as an illustration. Lane 1, M13 genome Control. Lane 2, M13 genome contain 58mer AG*C ligation product (G*=dG-C8-AAF).

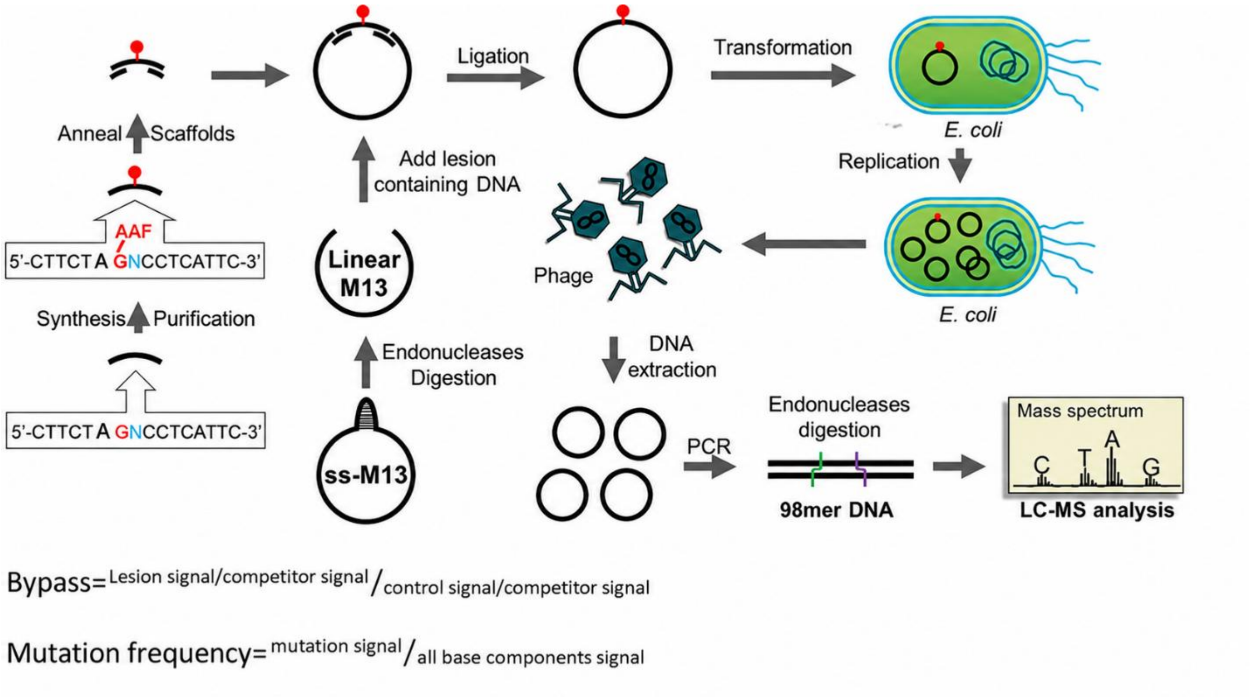

Figure S21. Diagram of the CRAB & REAP procedures using AG*N as an illustration (G*=dG-C8-AAF).

Table S1. Calculated and observed monoisotopic MW and m/z value of modified oligonucleotides. (G*=dG-C8-AAF).

5'-CTTCT**A/TG*N** CCTCATTC-3'	MW (calculated) of neutral species	m/z (calculated) -3 charge peak	m/z (observed) -3 charge peak
16mer AG*C	4961.8969	1652.9578	1652.9432
16mer TG*C	4952.8853	1649.9540	1649.9491
16mer AG*T	4976.8966	1657.9577	1657.9620
16mer TG*T	4967.8850	1654.9538	1654.9596
16mer AG*A	4985.9081	1660.9616	1660.9549
16mer TG*A	4976.8966	1657.9577	1657.9532
16mer AG*G	5001.90306	1666.2932	1666.2882
16mer TG*G	4992.8915	1663.2893	1663.2925

Table S2 Calculated and observed monoisotopic mass values and m/z values measured by MALDI-TOF.

Sequence	Calculated Monoisotopic Mass		Actual m/z	
	Full Length (Da)	Digested (Da)	Full Length	Digested
AGC-AAF	4962.90	2292.48	4966.79	2291.31
TGC-AAF	4953.89	2283.47	4954.67	2288.85
AGT-AAF	4977.90	2292.48	4976.60	2290.67
TGT-AAF	4968.89	2283.47	4970.02	2283.37
AGA-AAF	4986.92	2292.48	4982.50	2291.75
TGA-AAF	4977.90	2283.47	4973.53	2282.68
AGG-AAF	5002.91	2292.48	5002.89	2292.38
TGG-AAF	4993.90	2283.47	4991.07	2283.74

Table S3. List of oligonucleotide and primer sequences (5’ to 3’) used for the CRAB and REAP assays.

AGC	AGC Control	CACTTCTAGCCCTCATTCTC
	AGC Control 3’end oligo	TACCGTCGCTACGCGCATGCA
	AGC Control 5’end oligo	TCTCGAGTGCATCGTCAGCAC
	AG*C	CTTCTAG*CCCTCATTC
	AG*C 3’end oligo	TACCGTCGCGGCGCGCATGCA
	AG*C 5’end oligo	TCTCGAGTGACTCGTCAGCAC
AGT	AGT Control	CTTCTAGTCCTCATTC
	AGT Control 3’end oligo	TACCGTCGCTACGCGCATGCA
	AGT Control 5’end oligo	TCTCGAGTGATGCGTCAGCAC
	AG*T	CTTCTAG*TCCTCATTC
	AG*T 3’end oligo	TACCGTCGTAGCGCGCATGCA
	AG*T 5’end oligo	TCTCGAGTGATGCGTCAGCAC
AGA	AGA Control	CTTCTAGACCTCATTC
	AGA Control 3’end oligo	TACCGTCGGAACGCGCATGCA
	AGA Control 5’end oligo	TCTCGAGTGACTCGTCAGCAC
	AG*A	CTTCTAG*ACCTCATTC
	AG*A 3’end oligo	TACCGTCGCATCGCGCATGCA
	AG*A 5’end oligo	TCTCGAGTGCATCGTCAGCAC

AGG	AGG Control	CTTCTAGGCCTCATTC
	AGG Control 3'end oligo	TACCGTCGCTACGCGCATGCA
	AGG Control 5'end oligo	TCTCGAGTGTAGCGTCAGCAC
	AG*G	CTTCTAG*GCCTCATTC
	AG*G 3'end oligo	TACCGTCGACTCGCGCATGCA
	AG*G 5'end oligo	TCTCGAGTGCATCGTCAGCAC
TGC	TGC Control	CACTTCTTGCCCTCATTCTC
	TGC Control 3'end oligo	TACCGTCGGTTCGCGCATGCA
	TGC Control 5'end oligo	TCTCGAGTGATGCGTCAGCAC
	TG*C	CTTCTTG*CCCTCATTC
	TG*C 3'end oligo	TACCGTCGAACCGCGCATGCA
	TG*C 5'end oligo	TCTCGAGTGATGCGTCAGCAC
TGT	TGT Control	CTTCTTGTCCTCATTC
	TGT Control 3'end oligo	TACCGTCGGTTCGCGCATGCA
	TGT Control 5'end oligo	TCTCGAGTGTAGCGTCAGCAC
	TG*T	CTTCTTG*TCCTCATTC
	TG*T 3'end oligo	TACCGTCGTCACGCGCATGCA
	TG*T 5'end oligo	TCTCGAGTGATGCGTCAGCAC
TGA	TGA Control	CTTCTTGACCTCATTC
	TGA Control 3'end oligo	TACCGTCGAGACGCGCATGCA

	TGA Control 5'end oligo	TCTCGAGTGACTCGTCAGCAC
	TG*A	CTTCTTG*ACCTCATTC
	TG*A 3'end oligo	TACCGTCGCTACGCGCATGCA
	TG*A 5'end oligo	TCTCGAGTGACTCGTCAGCAC
TGG	TGG Control	CTTCTTGGCCTCATTC
	TGG Control 3'end oligo	TACCGTCGTGTCGCGCATGCA
	TGG Control 5'end oligo	TCTCGAGTGACTCGTCAGCAC
	TG*G	CTTCTTG*GCCTCATTC
	TG*G 3'end oligo	TACCGTCGGTTCGCGCATGCA
	TG*G 5'end oligo	TCTCGAGTGCGGCGTCAGCAC
AAF Competitor	19mer	CTTCTTGACCTCATTCTAG
	19mer 3'end oligo	TACCGTCGCTACGCGCATGCA
	19mer 5'end oligo	TCTCGAGTGTGTCGTCAGCAC
εA	εA Control	GAAGACCTAGGCGTCC
	εA Control 3'end oligo	TACCGTCGTTCCGCGCATGCA
	εA Control 5'end oligo	TCTCGAGTGAACCGTCAGCAC
	εA	GAAGACCTA*GGCGTCC
	εA 3'end oligo	TACCGTCGTAGCGCGCATGCA
	εA 5'end oligo	TCTCGAGTGAACCGTCAGCAC

	εA Competitor	GAAGACCTAGGCGTCCTAG
	εA Competitor 3’end oligo	TACCGTCGATGCGCGCATGCA
	εA Competitor 5’end oligo	TCTCGAGTGAACCGTCAGCAC
	Scaffold I	AGAAGTGCATGCGCG
	Scaffold II	CACTCGAGAGAATGAGG
	Scaffold III	CGACGGTACACTGAATCATGGTCATAGC
	Scaffold IV	AAAACGACGGCCAGTGAATTGTGCTGACG
	190 primer Forward	TTGTGTGGAATTGTGAGCGG
	190 primer Reverse	TGCAAGGCGATTAAGTTGGG
	230 primer Forward	CACCCCAGGCTTTACACTTT
	230 primer Reverse	GCAAGGCGATTAAGTTGGGTAA
	MS Forward primer	CGCCAGGGTTTTCCCAGTCACGAC
	MS Reverse primer	AGCGGATAACAATTTCACACAGGA

Table S4. Results of bypass (CRAB assay) of dG-AAF in HK82 *E. coli* cell (AlkB-). The bypass efficiency is in %.

	C	T	A	G
AG*N	6.49±0.25	6.38±0.89	8.16±0.44	9.69±0.05
AG*N	50.37±2.03	24.81±0.93	23.64±0.35	35.83±0.6

Table S5. Results of mutagenicity (REAP assay) of dG-AAF in HK82 *E. coli* cell (AlkB-). The mutation ratio was presented as in %. The total mutation of a certain sequence was calculated as the total of G>A, G>C, and G>T.

	C	T	A	G	-G	Total mutation
AG*C	0.15±0.05	0.36±0.03	5.98±2.38	93.51±2.45	N.D.	6.5±2.5
TG*C	0.19±0.02	0.08±0.02	0.82±0.41	98.92±0.39	N.D.	1.1±0.4
AG*T	0.10±0.04	0.18±0.03	7.93±1.90	91.79±1.90	N.D.	8.2±1.9
TG*T	0.21±0.18	0.12±0.01	2.11±0.56	97.56±0.74	N.D.	2.4±0.7
AG*A	0.10±0.04	0.35±0.28	0.93±0.18	98.61±0.39	N.D.	1.4±0.4
TG*A	0.20±0.05	0.23±0.12	0.69±0.04	98.88±0.16	N.D.	1.1±0.2
AG*G	0.47±0.4	1.88±0.38	1.60±0.41	96.05±1.16	N.D.	4.0±1.2
TG*G	0.14±0.01	0.18±0.04	0.56±0.01	99.11±0.02	N.D.	0.9±0.0

N.D.: not detected.

Table S6. Statistical analyses of bypass efficiencies. The analyses were conducted by comparing the bypass efficiency in percentage (Table S4) of a certain sequence to either inside a context group (such as TGN or AGN) or to the corresponding sequence in the other sequence group (such as TGC vs AGC). The analyses were performed using IBM SPSS Statistics. Differences in bypass efficiency among sequence contexts were assessed using Welch's one-way ANOVA, followed by Games–Howell post hoc multiple-comparison tests. All tests were two-sided, and p values < 0.05 were considered statistically significant. ns: $p > 0.05$; * $p < 0.05$, ** $p < 0.01$, *** $p < 0.001$.

	TGC	TGT	TGA	TGG	AGC	AGT	AGA	AGG
**TGC**	—	p < 0.01 **	p < 0.01**	p < 0.05*	p < 0.01**			
**TGT**		—	ns	p < 0.001***		<0.001 ***		
**TGA**			—	p < 0.001***			p < 0.001***	
**TGG**				—				p < 0.001***
**AGC**					—	ns	ns	p < 0.01**
**AGT**						—	ns	ns
**AGA**							—	ns
**AGG**								—

Table S7. Statistical analyses of total mutations. The total mutation in percentage (Table S5) means the total of G>A, G>T, and G>C mutations. The analyses were conducted by comparing the total mutation of a certain sequence to either inside a context group (such as TGN or AGN) or to the corresponding sequence in the other sequence group (such as TGC vs AGC). The analyses were performed using IBM SPSS Statistics. Differences in mutation among sequence contexts were assessed using Welch's one-way ANOVA, followed by Games–Howell post hoc multiple-comparison tests. All tests were two-sided, and p values < 0.05 were considered statistically significant. ns: p > 0.05.

	**TGC**	**TGT**	**TGA**	**TGG**	**AGC**	**AGT**	**AGA**	**AGG**
**TGC**	—	ns	ns	ns	ns			
**TGT**		—	ns	ns		ns		
**TGA**			—	ns			ns	
**TGG**				—				ns
**AGC**					—	ns	ns	ns
**AGT**						—	ns	ns
**AGA**							—	ns
**AGG**								—
